# Deposition of Metal-Organic Frameworks by Liquid-Phase Epitaxy: The Influence of Substrate Functional Group Density on Film Orientation

**DOI:** 10.3390/ma5091581

**Published:** 2012-09-05

**Authors:** Jinxuan Liu, Osama Shekhah, Xia Stammer, Hasan K. Arslan, Bo Liu, Björn Schüpbach, Andreas Terfort, Christof Wöll

**Affiliations:** 1 Institute of Functional Interfaces, Karlsruhe Institute of Technology, Hermann-von-Helmholty-Platz 1, B 330, Eggenstein-Leopoldshafen D-76344, Germany; E-Mails: jinxuan.liu@kit.edu (J.L.); xia.stammer@kit.edu (X.S.); hasan.arslan@kit.edu (H.K.A.); 2 Advanced Membranes & Porous Materials Center, King Abdullah University of Science and Technology, Thuwal 23955-6900, Saudi Arabia; E-Mail: osama.shekhah@kaust.edu.sa; 3 Department of Chemistry, University of Liverpool, Crown Street, Liverpool L69 7ZD, UK; E-Mail: bo.liu@liverpool.ac.uk; 4 Institute of Inorganic and Analytical Chemistry, Goethe-University Frankfurt am Main, Frankfurt 60438, Germany; E-Mails: Bjoern.Schuepbach@gmx.net (B.S.); aterfort@chemie.uni-frankfurt.de (A.T.)

**Keywords:** HKUST-1, SAMs, MOF thin film

## Abstract

The liquid phase epitaxy (LPE) of the metal-organic framework (MOF) HKUST-1 has been studied for three different COOH-terminated templating organic surfaces prepared by the adsorption of self-assembled monolayers (SAMs) on gold substrates. Three different SAMs were used, mercaptohexadecanoic acid (MHDA), 4’-carboxyterphenyl-4-methanethiol (TPMTA) and 9-carboxy-10-(mercaptomethyl)triptycene (CMMT). The XRD data demonstrate that highly oriented HKUST-1 SURMOFs with an orientation along the (100) direction was obtained on MHDA-SAMs. In the case of the TPMTA-SAM, the quality of the deposited SURMOF films was found to be substantially inferior. Surprisingly, for the CMMT-SAMs, a different growth direction was obtained; XRD data reveal the deposition of highly oriented HKUST-1 SURMOFs grown along the (111) direction.

## 1. Introduction

Metal-organic frameworks (MOFs) [1,2,3,4] are highly crystalline porous coordination polymers (PCPs) consisting of two essential building components: metal or metal/oxo coupling units and functional organic linkers. This new class of materials has attracted a lot of interest due to its enormous potential for applications in gas storage [4], gas separation [5,6], catalysis [7,8,9,10,11], drug delivery [3,12], sensors [13,14,15,16], electronic devices [17,18,19], light emitting devices [20], and as anode in Li-based batteries [21]. For a number of important applications, MOF thin films [22,23,24] are of special importance in particular with regard to electrochemistry [25], sensor technology [26], in electronic devices [27] as well as biocompatible substrates [28].

Because of the importance of fabricating well-defined, stable MOF coatings, in recent years numerous different approaches have been described, which have been recently reviewed [23]. Bein and coworkers [29] reported that immersion of organic templates into mother solutions (mother liqueur) of HKUST-1 yields HKUST-1 thin films consisting of highly oriented crystallites orientated with their (100) direction perpendicular to the substrate in case of COOH terminated self-assembled monolayers (SAMs), whereas for an OH terminated SAM the crystallites were found to align their (111) direction perpendicular to the substrate surface. Interestingly, Zacher *et al*. found a different orientation when MOF films were grown on oxidic substrates by immersion into solutions containing HKUST-1 crystallites [30]. A particular important approach with regard to the fabrication of highly orientated MOF coatings is the liquid-phase epitaxy (LPE) process which has been introduced by Shekhah *et al*. [31,32,33,34]. The LPE method has a number of advantages over other MOF deposition schemes, including a well-defined thickness, a high degree of orientation within the deposited films and the absence of pin-hole defects [25,31,32,33,34]. An important prerequisite for the epitaxial growth of SURMOFs (surface-mounted metal-organic frameworks) via the LPE method is the use of a templating substrate. In the majority of previous work on MOF thin film deposition using the LPE method, organic surfaces exposed by self-assembled monolayers, SAMs [35], have been used to provide organic surfaces which in turn act as nucleation sites for the epitaxial growth. It has been demonstrated previously that the choice of the SAM is quite crucial, e.g., on a CH_3_-terminated SAM MOF growth is completely suppressed [33]. Choosing a well-suited organic function to be attached at the end (ω-position) of an organothiol (with the SH-group at the α-position) makes a deposition not only possible, but also controls the crystallographic orientation of the deposited MOF film [34].

It has been reported that HKUST-1 ([Cu_3_BTC_2_·xH_2_O]_n_, btc = benzene-1,3,5-tricarboxylate) grows along the (100) direction on a carboxylic acid-terminated MHDA (16-mercaptohexadecanoic acid) SAM, whereas on a hydroxyl-terminated MUD (16-mercaptoundecan-1-ol) SAM or pyridine-terminated PPMT ((4-(4-pyridyl)phenyl)methanethiol) SAM, a different growth direction, (111), is obtained [29,31,34,36].

In this contribution, we report a systematic study on the influence of the packing density of the carboxylic acid group on the orientation and quality of MOF thin films grown using the liquid phase epitaxy method [31,32].

**Figure 1:**
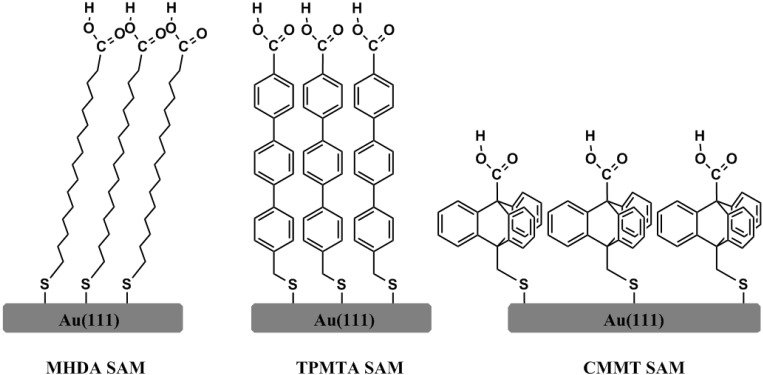
Schematic drawings of various carboxylic groups-terminated self-assembled monolayers (SAMs).

The area density of carboxylic acid groups exposed at the surface is varied by choosing organothiols with different molecular backbones that is alkane, oligophenyl, and triptycene as shown in Figure 1. We will demonstrate that, somewhat unexpectedly, the packing density of COOH-groups not only controls the quality of the MOF films but that there is also an area density threshold above which a switch of the growth direction of the SURMOF is observed. This observation provides important information about the growth mechanism of MOFs in the context of the LPE method.

## 2. Experimental Section

### 2.1. SAMs Preparation

The preparations of the carboxylic groups-terminated SAMs have been described in detail in earlier studies [37,38]. Briefly, the fabrication process involves immersion of the Au substrates into ethanolic solutions of 16-mercaptohexadecanoic acid (MHDA, Aldrich, 97%), *4*-mercaptomethyl-*4”*- terphenylcarboxylic acid (TPMTA) and 9-carboxy-10-(mercaptomethyl)triptycene (CMMT) [39]. During the preparation of MHDA- and TPMTA-SAMs, a small amount of acetic acid was added to the ethanolic solutions in order to obtain well-ordered SAMs by avoiding formation of hydrogen-bonds between neighboring carboxylic groups [37]. In case of the CMMT-SAMs, the ethanolic solution was used without adding additional acid.

### 2.2. MOF Thin Films Preparation

To study the growth behavior of MOF thin films on the different types of carboxylic acid terminated SAMs, we have chosen HKUST-1 as a model system. The experimental procedure used to grow HKUST-1 on organic surface has been discussed in some detail previously [29,31,32,33,34,40]. In short, for HKUST-1 the epitaxial growth process consists of alternately immersing an appropriate SAM substrate into ethanolic solutions of the building units: copper acetate and H_3_BTC (benzene-1,3,5-tricarboxylic acid), Between each immersion step the substrates are rinsed thoroughly with ethanol. In the present work the SAM substrates were immersed into 1 mM ethanolic solution of copper acetate for 15 min, subsequently rinsed with pure ethanol solution for 2 min, and then immersed into H_3_BTC solutions for 30 min. All the solutions were kept at 50 °C during MOF thin film preparations.

### 2.3. X-ray Diffraction (XRD)

XRD was carried out on Bruker D8 Advance in θ–2θ geometry equipped with a Si-strip detector (PSD Lynxeye (C)) using Cu K_alpha1,2 radiation. On the tube side a variable divergence slit set to V12 (fixed slit with 12 mm opening) and on the receiving side a 2.5° Soller slit was used.

Scans ran from 5° to 20° (2θ) with a step width of 0.024° and 2 seconds per step, which resulted in a total step counting time of 178 seconds due to the specific PSD settings.

Evaluation of data was done with Bruker evaluation software EVA 15.0. After background correction the peak position were calibrated using the position of the substrate Au(111) diffraction peak position, which was measured additionally at the end of each run.

### 2.4. Infrared (IR) Spectroscopy

The IRRA spectrum of the CMMT-SAMs was acquired with a resolution of 2 cm^−1^ using a FTIR spectrometer (Bruker VERTEX 80v) attached to a UHV apparatus (Prevac) with a base pressure of the measurement chamber of 2 × 10^−10^ mbar [41]. All the IRRA spectra were recorded in grazing incidence reflection mode at an angle of incidence amounting to 80° relative to the surface normal using liquid nitrogen cooled mercury cadmium telluride (MCT) narrow band detectors. The spectra of MHDA and TPMTA-SAMs were taken under ambient conditions with a Bruker VERTEX 70 FTIR spectrometer. Perdeuterated hexadecanethiol-SAMs on Au/Si were used for reference measurements.

## 3. Results and Discussion

**Figure 2:**
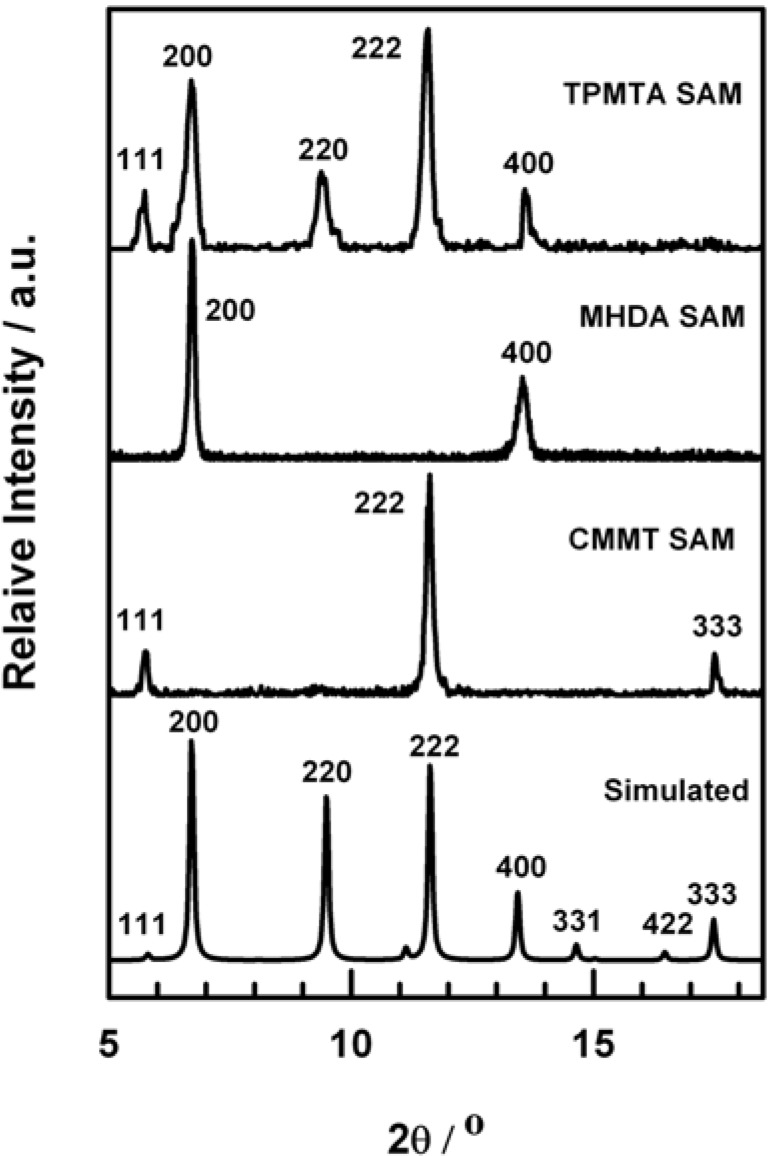
X-ray diffraction patterns (background corrected) thin films of HKUST-1 on various carboxylic groups functionalized gold surfaces after 40 deposition cycles. The patterns are compared with a simulated powder reference sample. Each pattern is normalized to the most intense reflection.

X-ray diffraction data of HKUST-1 films grown on MHDA-, TPMTA- and CMMT-SAMs, respectively, are shown in Figure 2. The XRD data demonstrates the presence of thin films of HKUST-1 on the different types of COOH-SAMs. The results for the SURMOF grown on the MHDA-SAM are consistent with previous findings, which reported a preferred growth along the (100) direction [31]. For the CMMT-SAM the HKUST-1 SURMOFs are also of high quality but, interestingly, show a different orientation (111). In case of the TPMTA-SAM the data reveal the presence of a low-quality SURMOF not exhibiting a well-defined growth direction but instead the presence of crystallites with mixed orientations, (100) and (111). These findings are somewhat surprising, in previous works it has been tacitly assumed that only the nature of the functionalities exposed at the SAM surface influence the orientation and the quality of the SURMOF, the but not their density [34,36].

From the full width at half maximum (FWHM) of the XRD peaks the thickness of the deposited MOF films can be calculated using the Scherrer equation [42]. In Figure 2, by evaluation of the FWHM of (200) and (222) peaks obtained for the HKUST-1 films on CMMT- and MHDA-SAMs substrates, thicknesses of 80 ± 5 nm and 41 ± 5 nm, respectively, are obtained after 40 growth cycles. These values yield a thickness per deposition cycle of 1 ± 0.1 nm along the (100) directions (MHDA) and of 2 ± 0.1 nm along the (111)-direction (CMMT). In the case of the thin film of HKUST-1 grown on TPMTA SAM, the XRD pattern demonstrates that the thin film is polycrystalline, with the crystallites exhibiting mixed orientations. The analysis of the most intense (100) and (111) peaks with the Scherrer equation yields an average thickness of 48 ± 5 nm for HKUST-1 grown on a TPMTA SAM.

**Figure 3:**
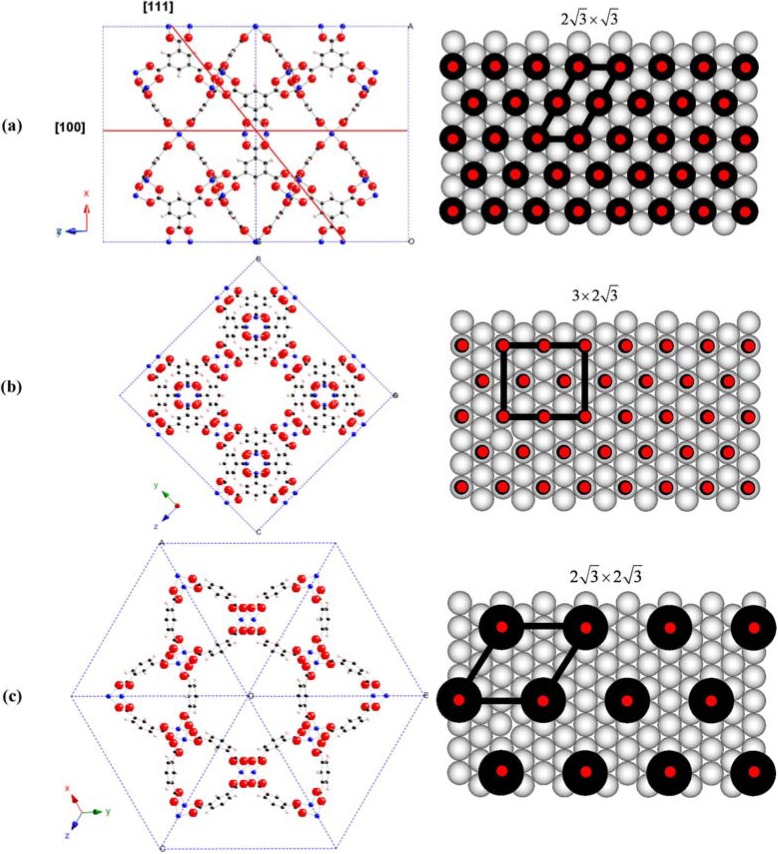
[Cu_3_(btc)_2_]_n_ (HKUST-1) metal-organic framework. The blue dashed line makes the unit cell; the bold red lines indicate the proposed contact planes between the SURMOF and the SAM. The black solid circles indicate the alkane and triptycene chains of the mercaptohexadecanoic acid (MHDA)- and 9-carboxy-10-(mercaptomethyl)triptycene (CMMT)-SAMs, respectively. The red solid circles indicate assumed positions of the carboxyl groups in the MHDA-, 4’-carboxyterphenyl-4-methanethiol (TPMTA)- and CMMT-SAMs, respectively. (**a**) Side view of the (100) and (111) planes and the overlayer structure of TPMTA-SAM; (**b**) top view of the (100) plane and the overlayer structure of MHDA-SAM; (**c**) top view of the (111) plane and the overlayer structure of CMMT-SAM.

The per-cycle thickness increase on the MHDA-SAM is in good agreement with the results reported previously by Ocal and coworkers [43], who studied the growth of HKUST-1 on patterned MHDA-SAMs using the standard LPE deposition process with AFM (atomic force microscopy). In this previous work it was found that each LPE cycle leads to the deposition of 1.317 nm of HKUST-1 along the (100) direction [43], which corresponds to half of the unit cell as shown in Figure 3. Assuming a similar growth mechanism for the growth along the (111)-direction, we expect an increase in thickness of 2.281 nm per deposition cycle, in good agreement with the thickness per deposition cycle observed in the present study.

The packing density of TPMTA-, CMMT- and MHDA-SAMs is assumed to be equal to that of SAMs made from *p*-mercaptomethyl-*p*-terphenyl [44], 10-(mercaptomethyl)triptycene [39] and alkanethiols [45] at Au(111) surface since functional groups have relatively little effect on the structure of the film in the molecular backbones [46,47]. The packing density of alkanethiolates at Au(111) surface has been reported to adopt a $\left(3\times 2\sqrt{3}\right)$ unit cell with four molecules per unit cell [45]. It has been shown that SAMs prepared from *p*-mercaptomethyl-*p*-terphenyl on Au substrates adopt a $(2\sqrt{3}\times \sqrt{3})R30{}^{\circ}$ unit cell [44]. In previous STM-work on SAMs made from triptycene-based thiolates and selenolates on Au substrate, the molecular arrangement of 10-(mercaptomethyl)triptycene molecules on Au substrate have been found to adopt a $(2\sqrt{3}\times 2\sqrt{3})R30{}^{\circ}$ unit cell of on Au(111) substrates [39]. This larger unit cell and the corresponding larger area per thiolate unit in the CMMT-SAMs are consistent with the more bulky structure of the triptycene backbone.

Assuming an ideal SAM structure with the unit-cell parameters as provided above, the density of COOH groups exposed at the organic surfaces exposed by the different types of SAMs used in the experiments reported here can be calculated, the results are shown in Table 1. It is obvious that the packing density of carboxylic acid groups on the three SAM surfaces are in the order of MHDA = TPMTA > CMMT.

**Table 1:** Carboxylic acid packing density on the different types of SAMs.

SAMs	Unit cell	Area of unit cell (Å^2^)	Number of molecules per unit cell	Density (COOH groups per nm^2^)
MHDA	$\left(3\times 2\sqrt{3}\right)$	86.6	4	4.6
TPMTA	$(2\sqrt{3}\times \sqrt{3})R30{}^{\circ}$	43.3	2	4.6
CMMT	$(2\sqrt{3}\times 2\sqrt{3})R30{}^{\circ}$	86.2	1	1.2

**Figure 4:**
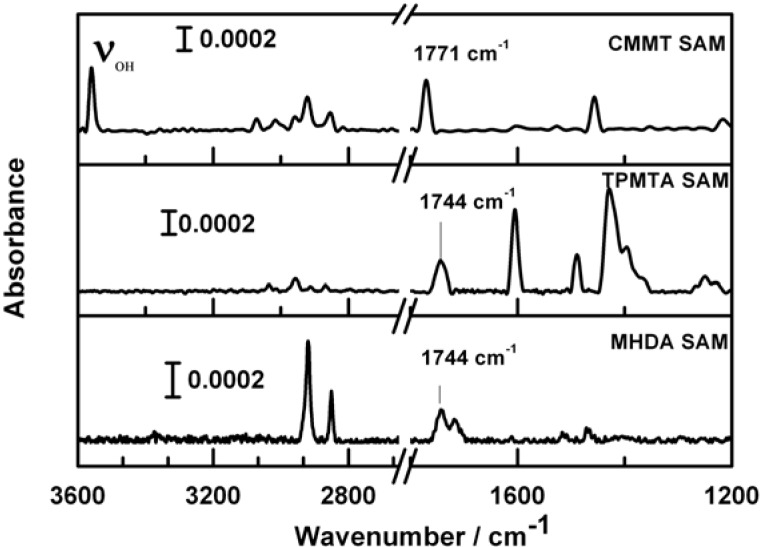
IRRA spectra of CMMT-, TPMTA- and MHDA-SAMs on gold.

The increased distance between the COOH-groups in the CMMT-SAMs is nicely corroborated by IRRAS-data showing the presence of a well-defined OH stretch vibration at around 3069 cm^−1^ and a single, sharp carbonyl stretch at 1771 cm^−1^ as shown in Figure 4. For the MHDA- and TPMTA-SAMs, it is known that no distinct OH-vibration was seen. This finding is attributed to a substantial broadening of the OH-stretch band, brought about by hydrogen-bonding between adjacent carboxylic acid groups. This explanation is corroborated by the fact that the corresponding carbonyl-vibrations is found to be substantially red-shifted to 1744 cm^−1^ for MHDA- and TPMTA-SAMs [37].

In previous work a detailed model for the growth mechanism of a HKUST-1 on carboxylic acid terminated surfaces has been proposed [34,36]. According to this model, the growth of HKUST-1 on a COOH-terminated HKUST-1 (100) surface proceeds as follows. First, the paddle-wheel Cu-acetates units [34] are bound to the exposed carboxylic acid units exposed at surface via the exchange of one (or two) acetate groups. In the next step, from the H_3_BTC-solution, BTC-units will bind to the surface by replacing the remaining paddle-wheel Cu-acetates units by the BTC carboxylates, again yielding a COOH-terminated HKUST-1 (100) surface. By repeating this LPE-cycle a well defined MOF thin film is obtained.

In this previous work it was assumed that reason for the (100)-direction realized in the LPE-process applied to COOH-terminated surfaces results from the fact that a (100)-plane cutting the HKUST-1 bulk structure at the position indicated in Figure 3 only contains carboxylate-groups. At first sight this assumption is not consistent with the present data showing that on a COOH-terminated surface with a lower density of COOH-groups a different growth direction, (111), is realized. A more careful inspection, however, reveals that the number of carboxylate units exposed at the CMMT-SAM surface, 1.2/nm^2^ as shown in Table 1, is substantially lower than that contained the shifted (100)-plane cutting through the bulk HKUST-1 structure (see Figure 4), 4.08/nm^2^. In fact, the number is much closer to the density of carboxylate-groups cut by the (111)-plane, 1.24/nm^2^ (see Figure 3). In the case of the TPMTA SAM, the density of COOH-groups (as judged from the size of the unit cell) is identical to that of the MHDA SAM (see Table 1). Nevertheless, the XRD data reveal the presence of crystallites with different orientations in the HKUST-1 SURMOF grown on the TPMTA SAM. This finding was reproduced several times. We speculate that the reason for this somewhat unexpected behavior is related to the higher rigidity of the TPMTA SAMs. It is conceivable that the higher flexibility of the alkyl backbones in the MHDA-SAMs (as compared to the rather rigid terphyenyl-backbones in the TPMTA SAMs) facilitates the anchoring of the HKUST-I MOF building units.

Based on this observation we propose that the growth direction of HKUST-1 prepared using the LPE-process is not determined by the precise type of surface functionalization but rather by the interphase energy between a given templating surface and the different oriented surfaces of a MOF-crystallite. Crystallites with low interface energy (or high binding energy) will be stabilized, while crystallites with different orientations will be destabilized and are prone to dissolution in the epitaxial process. Note, that when a HKUST-1 substrate terminated with Cu-acetate paddle wheels (see above) is exposed to a solution of carboxylic acids instead of the regular replacement of an acetate group by the carboxylic acid as required by the epitaxial growth process (see above) also a detachment of the Cu-acetate from the surface is possible. This process, which corresponds to dissolution of the substrate, competes with the deposition process.

We thus propose that the LPE-process favors crystallographic directions where the interface energy is lowest, e.g., in case of a good match of carboxylate packing motif within a bulk cutting-plane with the packing of carboxylic acids exposed on the organic surface used as a substrate for the epitaxial process.

## 4. Conclusions

Our systematic study on the epitaxial growth of HKUST-1 thin films on carboxylic acid terminated organic surfaces exposed by organothiolate-based SAMs demonstrates that highly oriented crystalline thin films can be grown along specific orientations. The growth direction can be controlled by the COOH functional group packing density, we obtain growth along the (100) direction on an MHDA SAM and along the (111) direction on a CMMT SAM. These findings suggest that the key factor determining the growth direction in MOF liquid phase epitaxy is the interface energy between the templating surface and the different crystallographic MOF-surfaces. Given the increasing importance of SURMOF thin films [48], theoretical work on the interfacial energies between MOF crystallites and organic surfaces as exposed by SAMs is urgently required.
